# Low-Baseline PD1+ Granulocytes Predict Responses to Atezolizumab–Bevacizumab in Hepatocellular Carcinoma

**DOI:** 10.3390/cancers15061661

**Published:** 2023-03-08

**Authors:** Catia Giovannini, Fabrizia Suzzi, Francesco Tovoli, Mariangela Bruccoleri, Mariarosaria Marseglia, Eleonora Alimenti, Francesca Fornari, Massimo Iavarone, Fabio Piscaglia, Laura Gramantieri

**Affiliations:** 1 Center for Applied Biomedical Research-CRBA, University of Bologna, IRCCS Azienda Ospedaliero-Universitaria di Bologna, 40138 Bologna, Italy; 2 Department of Medical and Surgical Sciences, Bologna University, 40138 Bologna, Italy; 3 Division of Internal Medicine, Hepatobiliary and Immunoallergic Diseases, IRCCS Azienda Ospedaliero-Universitaria di Bologna, 40138 Bologna, Italy; 4 Fondazione IRCCS Cà Granda Ospedale Maggiore Policlinico di Milano, Division of Gastroenterology and Hepatology Milan, 20122 Milan, Italy; 5 Department for Life Quality Studies, University of Bologna, 47921 Rimini, Italy

**Keywords:** HCC, immunotherapy, granulocytes, PD1, flow cytometry

## Abstract

**Simple Summary:**

Immune check point inhibitors (ICPIs) are one of the treatment options for advanced hepatocellular carcinoma (HCC). No biomarker is currently available to upfront select patients to be addressed to one or another drug. We have tested a prospective series of patients with advanced HCC treated with atezolizumab–bevacizumab combination with the aim of identifying biomarkers of response by using a simple cytofluorimetric test on peripheral blood. Due to the relevant role of granulocyte in the immune response, here we have focused on granulocyte immunophenotype by investigating PD1 and PD-L1 expression on their surface by using a simple cytofluorimetric test on peripheral blood. A low baseline PD1+ granulocyte percentage identified patients likely to benefit from atezolizumab–bevacizumab. Our findings warrant to validate this cheap and immediate cytofluorimetric test in larger cohorts, to verify whether the individual accuracy could be informative for the clinical practice.

**Abstract:**

Introduction: Immune check point inhibitors have recently entered the armamentarium of advanced hepatocellular carcinoma (HCC) treatment. Among them, the combination of atezolizumab plus bevacizumab has pushed it a step forward; however, a number of patients still present primary non-responses without any biomarker to predict responses to different options. Here, we aimed to identify a putative baseline biomarker to predict the response to atezolizumab–bevacizumab, by investigating whether baseline PD1+ and PD-L1+ peripheral granulocyte percentages might offer a non-invasive, cheap, and easily feasible assay. Methods: A prospective Italian cohort of 34 patients treated by atezolizumab–bevacizumab was tested to assay the baseline percentage of peripheral granulocytes and their PD1 and PD-L1 expression. The neutrophil to lymphocyte ratio (NLR) was also considered, and all data were compared with the clinical course of patients. Results: A low-baseline PD1+ peripheral granulocyte percentage turned out to predict responder patients (mean ±SD of PD1+ granulocyte percentage in responders versus non-responders: 9.9 ± 9.1 vs. 29.2 ± 17.6; student’s *t*-test, *p* < 0.01). In line, patients identified by a low PD1+ granulocyte percentage displayed a longer TTP (log-rank test, *p* < 0.0001). A lower granulocyte percentage on total white blood cells, irrespective of PD1 or PD-L1 expression, is also associated with responses to atezolizumab–bevacizumab (log-rank test, *p* < 0.05). No predictive value was observed for either the PD-L1+ granulocyte percentage or NLR. Conclusions: A low-baseline PD1+ peripheral granulocyte percentage is associated with responses to atezolizumab–bevacizumab treatment in advanced HCC. These findings encourage evaluating this minimally invasive, cheap, and easy test in further independent cohorts and outlining the relevance of innate immunity in the response to immune-checkpoint inhibitors.

## 1. Introduction

The treatment of advanced Hepatocellular Carcinoma (HCC) has recently improved due to the introduction of novel first- and second-line Tyrosine Kinase Inhibitors (TKIs), as well as immune checkpoint inhibitors. In the last decade, the PD1–PD-L1 axis was shown to inhibit the anti-cancer immune response, becoming a major therapeutic target. PD-L1 (Programmed Cell Death Ligand 1) is a trans-membrane protein expressed on the surface of many types of cancer cells as an adaptive immune mechanism. By binding to its ligand PD1 (Programmed Cell Death Protein 1), expressed by immune cells, PD-L1 activates resting programs by inhibiting lymphocytes proliferation and cytokine secretion and by inducing apoptosis [1]. The inhibition of PD1 and/or PD-L1 was shown to promote an effective immune response in subgroups of cancer patients, including patients with advanced HCC. In particular, the combination of atezolizumab plus bevacizumab (atezolizumab–bevacizumab) has moved treatment a step forward, and their combined use was recently licensed in Italy for the treatment of advanced HCC. A comprehensive study of the interactions between atezolizumab and PD-L1 and between PD1 and PD-L1 confirmed the overlap of the epitopes within the PD-L1 binding site, implying that atezolizumab blockades of PD-1/PD-L1 interactions occur by outcompeting PD-1 for binding to PD-L1. [2] On the other hand, bevacizumab is a recombinant humanized monoclonal antibody targeting VEGF (Vascular Endothelial Growth Factor), which plays a pivotal role in HCC development and progression by triggering neoangiogenesis as well as immune tolerance [3]. However, a number of patients still present primary non-responses. Mechanisms driving responses and resistance to atezolizumab–bevacizumab are under investigation, and some of them have been molecularly dissected at the tissue level [4].

The role of innate immunity and of non-lymphocytic populations in the modulation of responses to immune checkpoint inhibitors is known but still poorly understood in HCC [5]. Tumor-infiltrating lymphocytes are relevant in HCC, and their presence even identifies specific subtypes of HCC. In fact, according to the WHO’s classification, the lymphocyte-rich subtype portends a better prognosis [6] Similarly, the presence of neutrophil infiltration is relevant. Indeed, the neutrophil-rich subtype, despite being very rare, displays a much worse prognosis. A variable degree of infiltration of tumor tissue can be observed in a percentage of HCCs, and the few studies focused on neutrophil relevance have identified their presence as an adverse event, as reviewed by Arvanitakis K et al. [7]. Accordingly, in the peripheral blood, the neutrophil-to-lymphocyte ratio (NLR) shows a prognostic relevance in many tumor types, including HCC [8,9]. This also holds true in the setting of atezolizumab–bevacizumab treatment, where the NLR portends a predictive value [10]. Indeed, the pretreatment NLR was lower in patients who achieved disease control, while it was higher in patients who experienced disease progression. Even further, after three cycles of nivolumab treatment in advanced HCC patients, the NLR was associated with the response and longer overall survival, suggesting that eventual NLR changes early in the course of immune-based treatments might reflect the immune system’s activation by immunotherapy [11]. These findings were ascribed to the fact that the NLR is likely to reflect a general inflammatory profile which is deleterious for anti-cancer immune responses, even though the mechanisms were not defined. Phenotypic and transcriptomic changes occurring in tumor-infiltrating cells are under active investigation; conversely, less is known about circulating cells, which are, however, thought to participate in immune modulation too. Recent studies have focused attention on PD1’st role in the myeloid compartment as a key modulator of the T-cell immune response [12]. Interestingly, PD1 was shown to be highly expressed by granulocyte/macrophage progenitors emerging during cancer-driven “emergency myelopoiesis” as well as by tumor-infiltrating myeloid cells. Even more interestingly, Strauss et al. proved that myeloid-specific PD1 ablation in mice prevents the accumulation of granulocytic precursors and myeloid-derived suppressor cell (MDSC) generation and finally results in the inhibition of tumor growth by enhancing both the T-effector memory cells’ functionality and the antitumor T-cell response. In this study, myeloid cell-specific PD1 ablation was more effective than T-cell-specific PD1 ablation in decreasing tumor growth [12].

These findings open the way to the exploration of the non-lymphocytic compartment of circulating immune cells. Very limited data are available, to our knowledge, on PD1 and PD-L1 expression by peripheral non lymphocytic populations in HCC and particularly in the setting of immunotherapy, where a role of innate immunity can be envisaged. In the presence of malignancies, an increase in myeloid cell output occurs, as a response to immunologic stress. This phenomenon, called “emergency myelopoiesis”, is crucial because these innate immune cells activate adaptive immunity. A fraction of these expanded myeloid cells becomes MDSC, with immunosuppressive and tumor-promoting properties. These cells are associated with poor outcomes in many tumor types and inhibit responses to immunotherapy [13,14,15]. While MDSC have been investigated in HCC patients, less is known about peripheral granulocytes and their immunophenotype. Given their numeric prevalence, together with the ability to penetrate tissues, and especially cancer tissues, this cell population might also turn interesting in the setting of immunomodulating treatments.

Atezolizumab–bevacizumb treatment has become a front-line option in advanced HCC, due to the encouraging results of recent clinical trials, displaying a 30% response rate [16]. However, a wide proportion of patients do not respond to this treatment or escape from efficacy during treatment. Interestingly, novel approaches aimed at blocking the PD1-PD-L1 complex by oral small molecules or by PD-L1 B-cell peptide cancer vaccines able to stimulate the immune response are in advanced preclinical studies, pointing to the pivotal role of the PD1–PD-L1 axis in anticancer immune response [17,18]. Unfortunately, no biomarker is still available to predict the response to different ICPIs in patients with advanced HCC [19].

Here, we investigated the expression of PD1 and PD-L1 by circulating neutrophils in patients to be treated with atezolizumab–bevacizumab, to start elucidating their possible role as biomarkers. PD-L1 was chosen because it is the direct target of atezolizumab, while PD1 was chosen because it is a PD-L1 direct target and because interesting literature data converge on its role as a modulator of the lineage fate commitment and function of myeloid cells generated from tumor-driven emergency myelopoiesis [12].

## 2. Materials and Methods

### 2.1. Patients

The study cohort includes a total of 34 patients referred to S. Orsola-Malpighi University Hospital of Bologna and to the Center for Liver Disease, Maggiore Hospital, University of Milan, for the diagnosis and cure of advanced HCC (Table 1). Patients with intermediate or advanced HCC not amenable to curative treatments or TACE were evaluated for treatment with atezolizumab plus bevacizumab between April 2022 and August 2022. When no contraindication was identified, treatment was started with atezolizumab (1200 mg as an intravenous infusion) in association with bevacizumab (15 mg/kg IV) on the same day, every 3 weeks. Thirty-eight patients with intermediate or advanced HCC (BCLC B and C) according to the BCLC staging system [20] not amenable to potentially curative treatments or TACE were screened, and four of them did not receive the indication for the atezolizumab–bevacizumab treatment due to ongoing anticoagulant treatment (one case), previous liver transplantation (one case), high cardiovascular risk (one case), and venous leg ulcers (one case). Atezolizumab–bevacizumab treatment was performed according to a 3-week infusion schedule. Patients’ characteristics are reported in Table 1. All patients signed the informed consent form after being informed on the finality and modalities of the study. The study was conducted in accordance with the Declaration of Helsinki, and the study protocol was reviewed and approved by the local ethic committee (Comitato Etico Area Vasta Emilia Centro—AVEC) on 6 June 2021 (approval number 528/2021/Sper/AOUBo). Treatment allocation relied upon best clinical practices and local agency approval at the time of treatment choice.

Study tests consisted of cytofluorimetric quantification of CD45+, PD1+, and PD-L1+ peripheral granulocyte populations performed at baseline before treatment start. Data were prospectively collected and subsequently compared in patients experiencing response to treatments, stable disease, or progression, as assessed by imaging techniques, according to RECIST 1.1 criteria. The baseline imaging (TC or MR) was performed at the time of diagnosis of intermediate/advanced HCC, while the first imaging assessment (TC or MR) was performed 6–8 weeks after the first drug infusion and every 8–10 weeks thereafter. Tumor responses were evaluated according to RECIST 1.1 at every time point. For the purposes of this study, the analysis was focused on the first follow-up evaluation, while subsequent imaging assessments were used for time to progression (TTP) analyses. According to RECIST 1.1 criteria [21], the tumor burden was assessed on a maximum of five lesions and a maximum of two lesion per organ, which, in our patient population, is represented by the liver. Pathological lymph nodes were incorporated according to Eisenhauer EA et al. [21].

Disease progression was assessed in the case of an increase of at least a 20% in the sum of target lesions and a 5 mm absolute increase or the appearance of new lesions or neoplastic vascular thrombosis; partial responses were assessed in the case of an at least 30% decrease in the sum of diameters of target lesions with respect to the baseline sum diameters; stable disease was assessed when neither the criteria of disease progression nor those of partial responses were reached [21].Three patients in the long responder group became suitable for loco-regional treatments during the follow-up. Since we cannot dissect the contribution of these treatments to disease progression, these patients were removed from the TTP analysis. Another patient in the stable disease group (after the second imaging assessment) died due to a sepsis-complicating COVID-19 infection, and he was removed from the TTP analysis.

### 2.2. Analysis of Granulocyte Phenotype by Flow Cytometry

In the present study, attention was focused on granulocytes that were identified by using side scatter (SSC) versus CD45 flow cytometric plots as a validated method for distinguishing white blood cells [22]. Very recently, this approach was also used for the detection of leukocyte subtypes in patients with COVID-19 [23]. The SSC-versus-CD45 plots were evaluated by ensuring that CD45 was on the x axis and SSC on the y axis, and the plot dimensions were constant in all plots. Flow cytometry analysis was performed on peripheral blood collected in EDTA vials at baseline immediately before atezolizumab–bevacizumab infusion. A total of 100 µL of blood were incubated for 20 min at room temperature with the following fluorescent antibodies: anti-human CD45 antibody (clone REA747), anti-human PD1 (Clone REA1165), and anti-human PD-L1/CD274, (clone REA1197) all supplied by Miltenyi Biotec GmbH (Bologna, Italy). Red blood cell lysis was carried out before cytofluorimetric analyses by using VersaLyse buffer (Beckman Coulter. Milano, Italy). Antibody dilution was chosen after appropriate titration and isotypic controls were used to set negative gates. Flow cytometry analyses were performed by using Cytoflex S (BecKman Coulter) daily, checked with S calibration beads (CytoFLEX Daily QC Fluorospheres, B53230) to keep the setting and histogram uniform over time.

### 2.3. Statistical Analysis

Comparisons between PD1+ and PDL1+ granulocyte percentages in responder versus non-responder patients were performed by unpaired Student’s *t*-tests. Patients displaying a partial response and a stable disease were grouped together as “responders” because disease stability is considered a relevant aim of treatment in advanced HCC. To further dissect any difference in the analyzed parameters among patients displaying partial responses, stable disease, and progressive disease, ANOVA was used in specific settings.

To evaluate any association between the high and low percentage of PD1+ and PD-L1+ granulocytes and responses to treatment, patients were stratified into high and low sub-groups based on the mean percentage of PD1+ and PDL1+ granulocytes as a cut off. The Kaplan–Meier survival curves with log-rank tests were used to test differences in time to progression (TTP) during atezolizumab–bevacizumab treatment between patients displaying high- or low-baseline PD1+ and PD-L1+ percentages among circulating granulocytes. The Kaplan–Meier survival curves with log-rank tests were also used to test differences in TTP during treatment between patients displaying high or low-baseline granulocyte percentages and high or low neutrophil-to-lymphocyte ratios (NLR), both based on mean values. Significant events were the HCC progression documented at imaging. Four patients were excluded from TTP analysis due to a sepsis-related death in one case and to the performance of loco-regional treatments during follow-ups in three cases.

Pearson’s correlation was used to explore the relationship between PD1+ and PD-L1+ peripheral granulocyte percentages and AFP, NLR and neutrophil, eosinophil, and basophil counts.

Reported *p* values were two-sided and considered significant when lower than 0.05. Statistical calculations were executed using SPSS version 20.0 (SPSS inc). * *p* < 0.05; * *p* < 0.01; *** *p* < 0.001.

## 3. Results

### 3.1. Intersample and Interday Reproducibility

Even though flow cytometry is a consolidated technique, we verified whether our readings were confirmed in different blood samples from the same patient taken in the same day. Moreover, since granulocytes are short lived, with a circulating half-life of 6–8 h, blood samples from the same patient taken on different days in close proximity were also analyzed. Two patients were subjected to two different blood samplings on the same day, and three other patients were assayed on two different days, with a five-day interval, before immunotherapy started. As shown in Table 2, no relevant difference was observed in different blood samples on the same day as well as in tests performed on different days.

### 3.2. Low-Baseline PD1+ Granulocytes Predict Responses to Atezolizumab–Bevacizumb and a Longer TTP

Atezolizumab targets PD-L1 on cancer cells, on infiltrating tumor cells, as well as on circulating cells. In our cohort of 34 advanced HCC patients treated by atezolizumab–bevacizumab, 27 patients were responders, including 12 patients with a partial response, 14 patients with a stable disease and 1 pseudoprogressor, while the remaining 7 patients showed disease progression since the first CT assessment. No complete response was observed. Interruption of atezolizumab–bevacizumab treatment was due to an imaging-documented disease progression in all patients except for one case with a stable disease, who died from severe sepsis complicating a COVID-19 infection. Two patients experienced gastrointestinal bleeding which conditioned a delay of atezolizumab administration and a temporary suspension of bevacizumab. In one case, bevacizumab was definitely stopped after the second event of intestinal bleeding. Firstly, we assessed the baseline expression of PD1 and PD-L1 on circulating granulocytes in patients to be treated by atezolizumab–bevacizumab, and we observed a variable percentage of positive cells among total granulocytes: PD1+ granulocytes ranged from 1% to 62% (mean ± SD:13 ± 14.2), while PD-L1+ granulocytes ranged from 34% to 49% (mean ± SD:42.03 ± 4.02), both displaying a relevant heterogeneity across patients. Next, we investigated whether this heterogeneity was associated with treatment responses and clinical variables.

**Figure 1:**
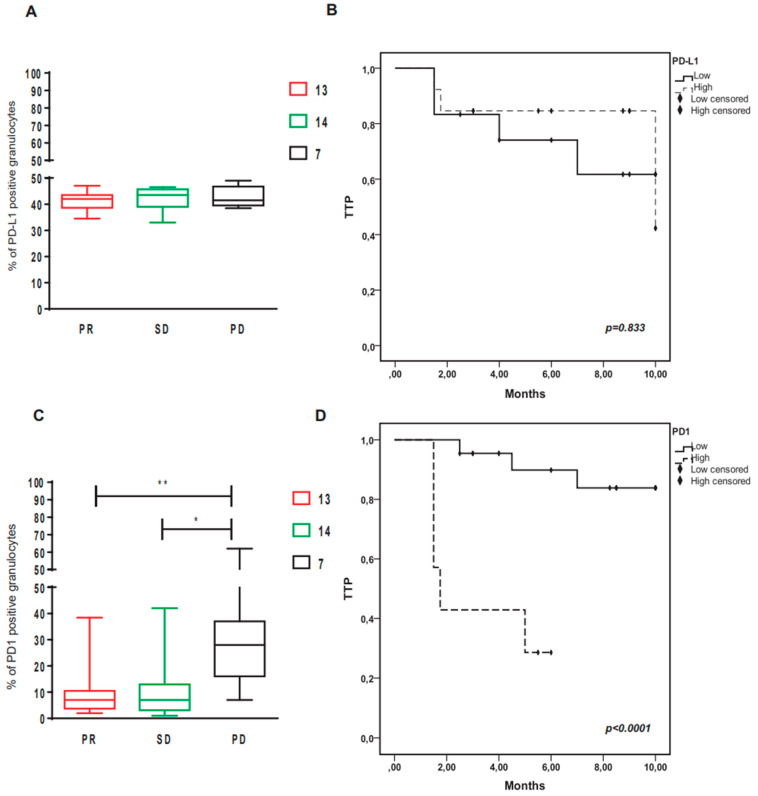
Baseline PD1+ and PD-L1+ granulocyte percentage in responders and non-responders to atezolizumab–bevacizumab: (**A**): box-plot graphic representation of PD-L1+ granulocyte percentage in patients displaying a partial response (PR; 13 patients), stable disease (SD; 14 patients), and progressive disease (PD; 7 patients) to atezolizumab–bevacizumab. (**B**): Kaplan–Meier survival curves comparing time to progression (TTP) in 34 patients treated with atezolizumab–bevacizumab. Patients were categorized as high or low PD-L1+ according to the mean value of PD-L1+ percentages of circulating granulocytes. Significant events were the HCC progression documented at imaging. Statistical *p*-values were generated by the log-rank test. (**C**): box-plot graphic representation of PD1+ circulating granulocyte percentage on total granulocytes in patients displaying a partial response (PR; 13 patients), stable disease (SD; 14 patients), and progressive disease (PD; 7 patients). * *p* < 0.05; ** *p* < 0.01 by ANOVA test. (**D**): Kaplan–Meier survival curves comparing TTP in 34 patients treated with atezolizumab–bevacizumab. Patients were categorized as high or low according to the mean value of PD1+ percentage of circulating granulocytes on total granulocytes, and statistical *p*-values were generated by the log-rank test. Significant events were the HCC progression documented at imaging.

The baseline percentage of PD-L1+ granulocytes on total granulocytes was not associated with a response or primary resistance to atezolizumab–bevacizumab (mean ± SD of PD-L1+ granulocyte percentage in responders versus non-responders: 41.5 ± 3.9 vs. 42.8 ± 4.1; student’s *t*-test: *p* = n.s.; Figure 1A).

Accordingly, when patients were separated based on the mean percentage value of PD-L1+ granulocytes, defining high or low groups, we did not observe any difference in time to progression (TTP) in patients displaying a different percentage of PD-L1+ granulocytes (Figure 1B).

Moving to PD1+ circulating granulocytes, the baseline percentage of this population turned out to be associated with the response to atezolizumab–bevacizumab: a lower percentage of PD1+ granulocytes predicted responses to treatment (mean ± SD of PD1+ granulocyte percentage in responders versus non-responders: 9.9 ± 9.1 vs. 29.2 ± 17.6; student’s *t*-test, *p* < 0.01; Figure 1C). We next compared the PD1+granulocyte percentage in patients displaying a partial response, stable disease, and progressive disease, confirming a significant difference of this biomarker in patients with disease progression (ANOVA: *p* < 0.01).

Moreover, when patients were separated according to the mean percentage of PD1+ granulocytes, a longer TTP could be observed in patients with a low PD1+ granulocyte percentage (log-rank test, *p* < 0.0001, Figure 1D). The mean ± SD follow-up was 3.2 ± 2.1 months in the 9 patients displaying a high PD1+ granulocyte percentage, while it was not reached in the 21 patients displaying a low PD1+ granulocyte percentage (less than 50% of patients had progression events during the follow-up period).

**Figure 2:**
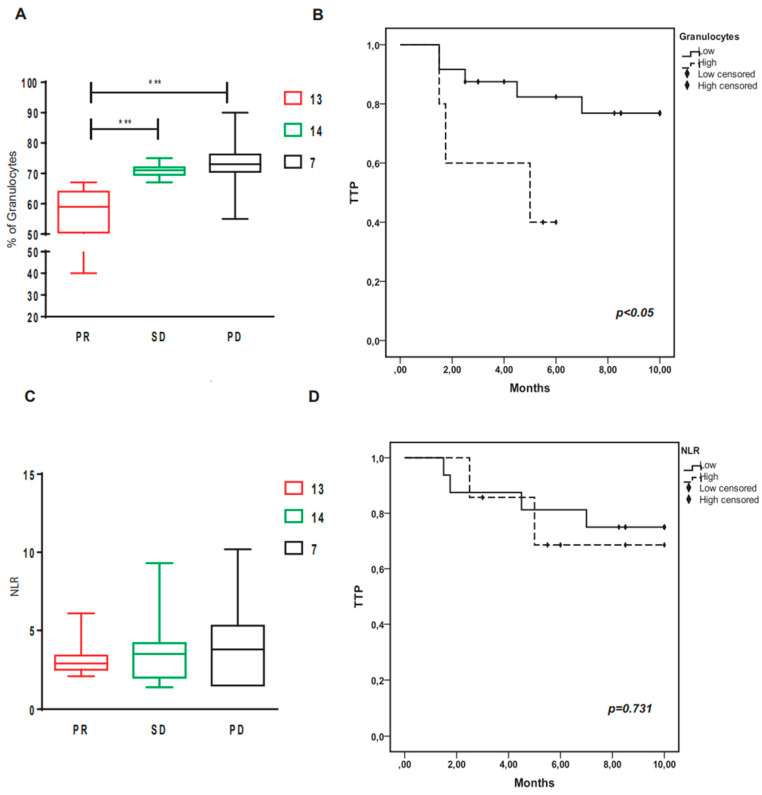
Baseline granulocyte percentage on total white blood cells and NLR in responders and non-responders to atezolizumab–bevacizumab: (**A**): box-plot graphic representation of granulocyte percentage in patients displaying a partial response (PR; 13 patients), stable disease (SD; 14 patients), and progressive disease (PD; 7 patients) to atezolizumab–bevacizumab. *** *p* < 0.001 by ANOVA test. (**B**): Kaplan–Meier survival curves comparing TTP in 34 patients treated with atezolizumab–bevacizumab displaying a high or low (according to mean values) circulating granulocyte percentage. Significant events were the HCC progression documented at imaging. Statistical *p*-values were generated by the log-rank test. (**C**): box-plot graphic representation of NLR in patients displaying a partial response (PR; 13 patients), stable disease (SD; 14 patients), and progressive disease (PD; 7 patients) to atezolizumab–bevacizumab. (**D**): Kaplan–Meier survival curves comparing TTP in 34 patients treated with atezolizumab–bevacizumab displaying high or low (according to mean values) NLR. Significant events were the HCC progression documented at imaging. Statistical *p*-values were generated by the log-rank test.

Then, we evaluated whether the granulocyte percentage on total white blood cells, irrespective of PD1 or PD-L1 expression, might be predictive of responses to treatment. A lower granulocyte percentage is associated with a partial response to atezolizumab–bevacizumab; however, the baseline granulocyte percentage is not informative in the prediction of stable disease, which is a relevant aim of immunotherapy in advanced HCC (Figure 2A).

In line, when patients were separated according to the mean percentage of granulocytes on total white blood cells, a longer time to progression for atezolizumab–bevacizumab treatment could be observed in patients with a lower granulocyte percentage, but, again, with a lower significance than that observed for PD1+ granulocytes (Figure 2B).

**Figure 3:**
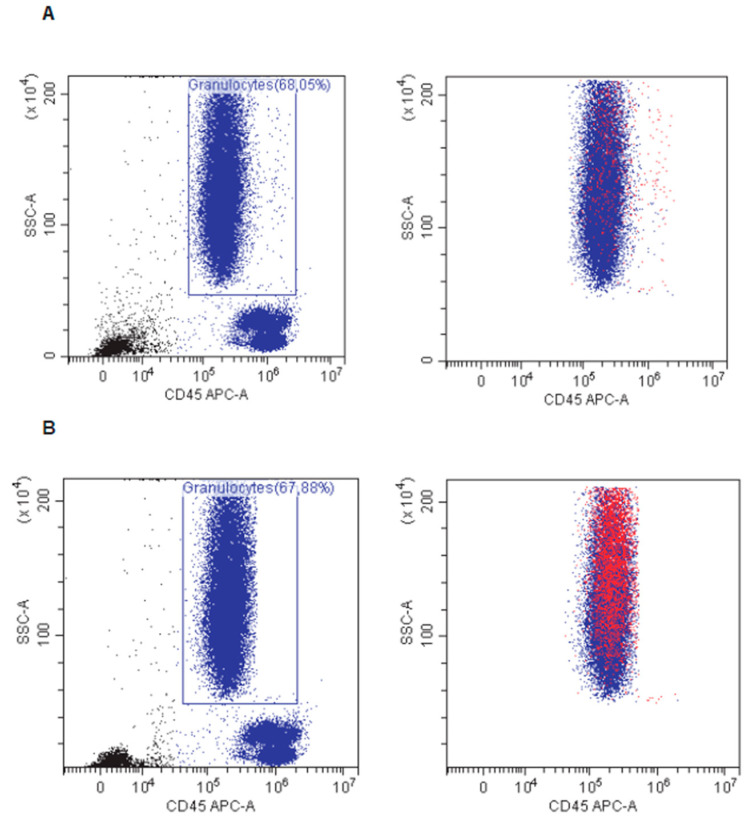
Representative dot plots showing PD1 expression on granulocytes evaluated by flow cytometry. Original dot plots from FACS analysis from two (**A**,**B**) representative patients are shown in the left panels displaying CD45 white blood cells. Right panels (**A**,**B**) focus on granulocytes only: PD1-negative granulocytes are represented in blue, while PD1-positive granulocytes are represented in red. The upper panels (**A**) are representative of patients displaying a low PD1-positive granulocyte percentage, while the lower panels (**B**) are representative of patients displaying a high PD1-positive granulocyte percentage.

Next, we tested possible correlations between PD1+ granulocytes and other laboratory variables including the PD-L1+ granulocyte percentage, total white blood cells, granulocyte populations (neutrophils, basophils, eosinophils), NLR, and AFP. The only significant correlation was found between the PD1+granulocyte percentage and NLR (Pearson’s correlation R = 0.58; *p* = 0.003), while a trend towards a negative correlation was observed between the PD1+granulocyte percentage and basophils (Pearson’s correlation R = −0.45; *p* = 0.05). When NLR was compared in responders and non-responders, no significant difference was found (Figure 2C). Accordingly, no difference was observed in TTP when patients were separated in high or low groups based on the mean value of NLR (Figure 2D). Taken together, our results showed that the PD1+ granulocyte percentage at baseline differs among patients (Figure 3), and lower values might predict a subgroup of patients likely to benefit from atezolizumab–bevacizumab.

## 4. Discussion

Neutrophils are the most abundant inflammatory cell population in peripheral blood. Despite the fact that they exert protective properties towards infections and some types of tumors due to their effector function in the innate immune response [24], their accumulation was reported to promote the progression of cancer and the metastatic process via several mechanisms, including neutrophil extracellular traps (NET) formation [25,26] and the inhibition of T-cell responses [27]. In HCC, both neutrophil accumulation and a high NLR were associated with a worse prognosis and displayed a predictive value in the setting of immunotherapy [6,7,8,9,28]. Expression of immune checkpoints on neutrophils has been mostly assessed in the intra-tumoral compartment. In gastric cancer and in HCC, their presence portends a prognostic significance [27,29]. Even though the expression and function of PD-L1 in tumor-infiltrating neutrophils still need to be fully elucidated, it was proposed to be crucial for the suppression of T-cell functions and for T-cell exhaustion [27]. Even stronger data support the relevance of PD1 expression on myeloid cells in the orchestration of responses to immune treatments. In an experimental setting, Strauss et al. showed that myeloid-specific PD1 ablation in mice prevents the accumulation of granulocytic precursors and MDSC and was more effective than T-cell-specific PD1 ablation in reducing tumor growth by enhancing the antitumor T-cell response [10]. Interestingly, promising therapeutic strategies leading to the abrogation of selected myeloid functions or neutrophil depletion are under investigation and might be preferentially utilized in defined patient subgroups [30]. Thus, beside their role as unspecific biomarkers of an inflammatory profile, neutrophils deserve investigation to unravel the specific mechanisms driving their prognostic and predictive roles, to control them in a therapeutic perspective. Indeed, several approaches for neutrophil antagonism are under investigation to potentiate immune checkpoint blockade [31]. In human studies, neutrophils are identified as CD66b+, CD15+ CD14−, and CD33+ white blood cells. Here, we have adopted an easy and simple cytofluorimetric approach, based on side scatter versus CD45 flow cytometric plots that allows for promptly recognizing granulocytes and rapidly identifying their PD1- and PD-L1-positive percentage [16,17]. This approach is less specific than the immunophenotypic-based identification of neutrophils; however, it allows for visualizing in the same scatter all the other blood cell populations such as lymphocytes and monocytes to be eventually compared and assessed as well. We did not aim to characterize the immunophenotype of specific granulocyte subpopulations. Instead, we aimed to evaluate whether circulating granulocytes express PD1 and PD-L1 and whether the expression of these molecules might be associated with responses to atezolizumab–bevacizumab. Indeed, lymphocytes are the most studied peripheral cell population in this setting, while very few data are available on granulocytes, even though preclinical findings point to their crucial role. [10,12,24,27]. Thus, PD1+ and PD-L1+ granulocyte percentages were quantified and tested as possible predictive biomarkers in the setting of atezolizumab–bevacizumab treatment in HCC patients. While PD-L1 was poorly informative, PD1 turned out to predict responses to treatment in our patient cohort. This was not unexpected, considering the basic research findings described above [12]. Even though the percentage of PD1+ granulocytes was the most informative parameter predicting the response to atezolizumab–bevacizumab, a lower granulocyte percentage on total white blood cells, irrespective of PD1 or PD-L1 expression, is also associated with responses to atezolizumab–bevacizumab, yet with a lower significance when compared to the PD1+ granulocyte percentage. In this exploratory study, we chose the mean percentage of PD1+ granulocytes as a cutoff value. Larger prospective series are under enrollment to accurately identify the most informative threshold value, also by assessing patients with different etiologies of the underlying chronic liver disease and different tumor stage. Conversely, in our cohort, the NLR, which considers neutrophils and lymphocytes only, did not show any predictive role. This might be ascribed to the small number of patients and to the high heterogeneity of NLR in non-responders.

## 5. Conclusions

Our findings, obtained from a well-annotated cohort of HCC patients, suggest that a simple test based on the PD1+ granulocyte percentage might predict subgroups of patients likely to benefit from atezolizumab–bevacizumab. In addition, they open the question as to whether the choice of specific ICPIs or their combinations might take advantage from similar specific tests. In particular, it might be interesting to test the PD1+ granulocyte percentage in patients undergoing PD1 blockade, and to assay whether inhibition of PD1 either alone or in association with anti-PD-L1 might prove more effective in patients with a high percentage of PD1+ granulocytes. In addition, since PD1 blockades induce a strong anti-tumor function by modulating an immunometabolic program of myeloid cells with a crucial role in systemic antitumor response, myeloid-specific PD-1 targeting was proposed as a tool to improve cancer immunotherapy. From this perspective, a simple and immediate test to assess patients with a higher PD1+ granulocyte percentage might give a possible stratifying marker.

## Figures and Tables

**Table 1:** Baseline demographics and clinical characteristics of patients treated with atezolizumab/bevacizumab.

Patient’s Characteristics		Atezolizumab+ Bevacizumab pts N = 34	Response to Treatment PR/SD/PD
Age (years old)	<65 years old ≥65 years old	10 (29.4%) 24 (70.6%)	4/3/3 9/11/4
Gender	M F	30 4	11/12/7 2/2/0
ECOG PS	0 1	28 (82.4%) 6 (17.6%)	12/10/7 1/4/0
Child-Pugh class	A B C	33 (97.1%) 1 (2.9%) 0	12/14/7 1/0/0 0
ALBI grade	1 2 3	20 (58.8%) 14 (41.2%) 0	7/10/3 6/4/4 0
BCLC stage	A B C	0 14 20	0 4/7/3 9/7/4
Etiology CLD	HBV active HCV cured HCV NASH/NAFLD alcohol	3 (8.8%) 3 (8.8%) 10 (29.4%) 15 (44.1%) 3 (8.8%)	0/1/2 2/1/0 6/2/2 3/9/3 2/1/0
Nodularity	uninodular multinodular ≤3 multinodular >3 infiltrating	9 5 10 10	4/3/2 1/3/1 4/4/2 4/4/2
Size (main lesion in multinodular)	≤3 cm 3–5 cm 5–10 cm >10 cm infiltrating (poorly defined)	7 8 10 7 2	
Portal vein invasion	yes no	14 (41.2%) 20 (58.8%)	6/4/4 7/10/3
Extrahepatic spread	yes no	11 (32.4%) 23 (67.6%)	4/4/3 9/10/4
AFP (ng/mL)	≤20 21–400 ≥401	15 (44.1%) 13 (38.2%) 6 (17.6%)	6/6/3 6/4/3 1/4/1
Response to treatment	partial response stable disease progression	13 14 7	

M: male; F: female; ECOG PS: Eastern Cooperative Oncology Group Performance Status (0–5); ALBI: Albumin-Bilirubin grade for HCC; PR/SD/PD: Partial Response/Stable Disease/Progressive Disease according to RECIST 1.1; BCLC: Barcelona Clinic Liver Cancer staging system; Etiology CLD: etiology of the underlying Chronic Liver Disease (CLD). In cases of more etiologies being recognized in the same patient, the most relevant was considered. HBV: Hepatitis B virus; HCV: Hepatitis C virus (active infection or previously cured infection); NASH/NAFLD: Non-Alcoholic Steato-Hepatitis/Non Alcoholic Fatty Liver Disease; AFP: Alfa-feto-Protein. Extrahepatic spread: lung: four cases (associated with cutaneous metastasis in one case), lymph nodes: four cases (associated with peritoneal metastasis in one case and with adrenal gland metastasis in another case), bone: two cases (associated with peritoneal metastasis in one case), brain: one case.

**Table 2:** Comparison of flow cytometric readings on granulocytes in different blood samples from the same patients obtained on the same day or on different days in proximity.

	Blood Sample	PD1+%	PDL1+%
Patient 1-T_0_	A	3%	44%
Patient 1-T_0_	B	3%	43%
Patient 2-T_0_	A	38%	41%
Patient 2-T_0_	B	40%	40%
Patient 3-T_0_	A	57%	41%
Patient 3-T_-1_	B	60%	39%
Patient 4-T_0_	A	15%	36%
Patient 4-T_-5_	B	16%	39%
Patient 5-T_0_	A	62%	49%
Patient 5-T_-5_	B	58%	46%

Five patients (#1 to #5) were tested by assaying two different blood samples (A and B). Blood samples from patients #1 and #2 were tested in the same day (T_0_, day of treatment start); blood samples from patient #3 were tested at treatment start (T_0_) and the day before (T_-1_); blood samples from patients #4 and #5 were tested at treatment start (T_0_) and 5 days before (T_-5_). Percentages of PD1+ and PD-L1+ granulocytes were calculated on total granulocytes.

## Data Availability

The data presented in this study are available on request to the corresponding authors.
